# A randomized controlled trial of a home-based computerized executive function intervention for children with cerebral palsy

**DOI:** 10.1007/s00431-023-05072-3

**Published:** 2023-07-18

**Authors:** María García-Galant, Montse Blasco, Olga Laporta-Hoyos, Alba Berenguer-González, Paula Moral-Salicrú, Júlia Ballester-Plané, Xavier Caldú, Júlia Miralbell, Xènia Alonso, Julita Medina-Cantillo, Elsa Povedano-Bulló, David Leiva, Roslyn N. Boyd, Roser Pueyo

**Affiliations:** 1 Departament de Psicologia Clínica i Psicobiologia, Universitat de Barcelona, Passeig de la Vall d’Hebron, 171, 08035 Barcelona, Spain; 2 Institut de Neurociències, Universitat de Barcelona, Passeig de la Vall d’Hebron, 171, 08035 Barcelona, Spain; 3 Institut de Recerca Sant Joan de Déu, Santa Rosa, 39-57, 08950 Esplugues de Llobregat, Barcelona, Spain; 4 Servei de Neurologia, Hospital Sant Joan de Déu, Passeig de Sant Joan de Déu, 2, 08950 Esplugues de Llobregat, Barcelona, Spain; 5 Servei de Rehabilitació i Medicina Física, Hospital Sant Joan de Déu, Passeig de Sant Joan de Déu, 2, 08035 Barcelona, Spain; 6 Departament de Psicologia Social i Psicologia Quantitativa, Universitat de Barcelona, Passeig de la Vall d’Hebron, 171, 08035 Barcelona, Spain; 7 Faculty of Medicine, Queensland Cerebral Palsy and Rehabilitation Research Centre, The University of Queensland, 62 Graham St, Brisbane, QLD 4101 Australia

**Keywords:** Cerebral palsy, Executive function, Computerized intervention, Cognitive intervention, Daily living

## Abstract

Children with cerebral palsy (CP) often show executive function (EF) impairments that are key to quality of life. The aim of this study was to assess whether a home-based computerized intervention program improves executive functions (EFs) compared to usual care. Sixty participants (30 females) with CP (8–12 years old) were paired by age, sex, motor ability, and intelligence quotient score and then randomized to intervention and waitlist control groups. The intervention group received a 12-week home-based computerized EF intervention (5 days/week, 30 min/day, total dose 30 h). Core and higher-order EFs were assessed before, immediately after, and 9 months after completing the intervention. The intervention group performed better than the waitlist control group in the three core EFs (immediately and 9 months after the intervention): inhibitory control (*F* = 7.58, *p* = 0.13 and *F* = 7.85, *p* = 0.12), working memory (*F* = 8.34, *p* = 0.14 and *F* = 7.55, *p* = 0.13), and cognitive flexibility (*F* = 4.87, *p* = 0.09 and *F* = 4.19, *p* = 0.08). No differences were found between the groups in higher-order EFs or EF manifestations in daily life.

*Conclusions*: A home-based computerized EF intervention improved core EFs in children with CP, but further research is needed to identify strategies that allow the transfer of these improvements to everyday life.

*Trial registration*: NCT04025749 retrospectively registered on 19 July 2019.

**Table:** 

**What is Known:** *• One in two children with cerebral palsy has an intellectual impairment. Visual perception and executive functions are the most reported specific cognitive deficits.* *• The majority of interventions for cerebral palsy focus on motor impairments, but only a few randomized controlled trials have explored the effect of interventions on executive functions.*	
**What is New:** *• A home-based computerized cognitive intervention can improve the core executive functions of children with cerebral palsy.* *• Short- and long-term effects on core executive functions have been found.*

**Supplementary Information:**

The online version contains supplementary material available at 10.1007/s00431-023-05072-3.

## Introduction

Cerebral palsy (CP) is a major cause of physical disability in children, with a median estimated prevalence of 1.6 per 1000 live births [1]. CP is described as a group of permanent disorders that affect the development of movement and posture, causing activity limitations attributed to nonprogressive disturbances that occur in the developing fetal or infant brain and persist throughout life [2]. This motor function impairment is often accompanied by disturbances of sensation, cognition, perception, communication, behavior, and epilepsy [2–4].

One in two children with CP has an intellectual impairment [5], which may be more disabling than the motor impairment itself [6, 7]. Visual perception [6] and executive functions (EFs) [8] are the most reported specific cognitive deficits in children with CP.

EFs include a set of complex cognitive skills that work together to direct behavior for decision-making and action planning and play a critical role in behavior and emotional control in daily life [9, 10]. EFs develop throughout childhood and adolescence and are essential for mental and physical health, daily life functioning, and academic achievement [9, 10].

According to Diamond [11], there are three core EFs: inhibitory control, working memory, and cognitive flexibility. Inhibitory control allows individuals to control their attention, behavior, thoughts, and/or emotions to override a strong internal predisposition or external lure. Working memory refers to the ability to hold information in mind and mentally process it. Cognitive flexibility is described as the ability to change perspectives or approaches to a problem and refers to flexibility in adjusting to new demands, rules, or priorities. These three core EFs constitute the fundamental base of higher-order EFs: reasoning, problem solving, and planning [11].

Difficulties in all three core EF domains (inhibitory control, working memory, and flexibility) are found in children with CP [7, 8, 12–18]. Furthermore, higher-order EF impairments have been reported in individuals with CP [7, 8, 13]. Some of the abovementioned studies used performance-based tests [8, 12–19], and others also used rating scales [7, 8, 15, 19], which are both necessary for the clinical diagnosis of neurological disorders, as they assess different aspects of EFs [9, 20, 21]. Considering that EF deficits have been identified in children with CP and the relationship between these deficits and the quality of life of people with CP [22], there is a clear need for EF interventions targeting people with CP.

The majority of interventions for CP focus on motor impairments [5, 23–25], but only a few randomized controlled trials (RCTs) have explored the effect of interventions (multimodal, physical, and cognitive) on EF. Specifically, some studies reported inhibitory control improvements immediately after multimodal [26], physical [19], or cognitive interventions [27]. Only a cognitive intervention showed efficacy in improving working memory immediately postintervention [27]. No previous short-term positive effects after multimodal, physical, or cognitive interventions on the core EF of cognitive flexibility were shown [28]. Only one multimodal study was previously conducted to determine the long-term follow-up effects on core EF outcomes. The results of that study (which did not include a control group) showed improvements in cognitive flexibility 6 months after the intervention [29]. Short-term positive effects of a multimodal intervention have only been found for the higher-order EF of reasoning [30]. Until now, there have been no studies targeting cognitive interventions that attempt to improve all core EF components with the same intervention, testing the effect on higher-order EFs and on daily life performance in children with CP. The present study was aimed at testing whether a home-based computerized cognitive intervention had positive short- and long-term effects on all core and higher-order EFs and manifestations of EF in daily life in children with CP.

## Materials and methods

### Study design and procedure

A researcher-blinded, matched-pair, randomized waitlist-controlled trial was performed as detailed in the study protocol [31].

**Fig. 1:**
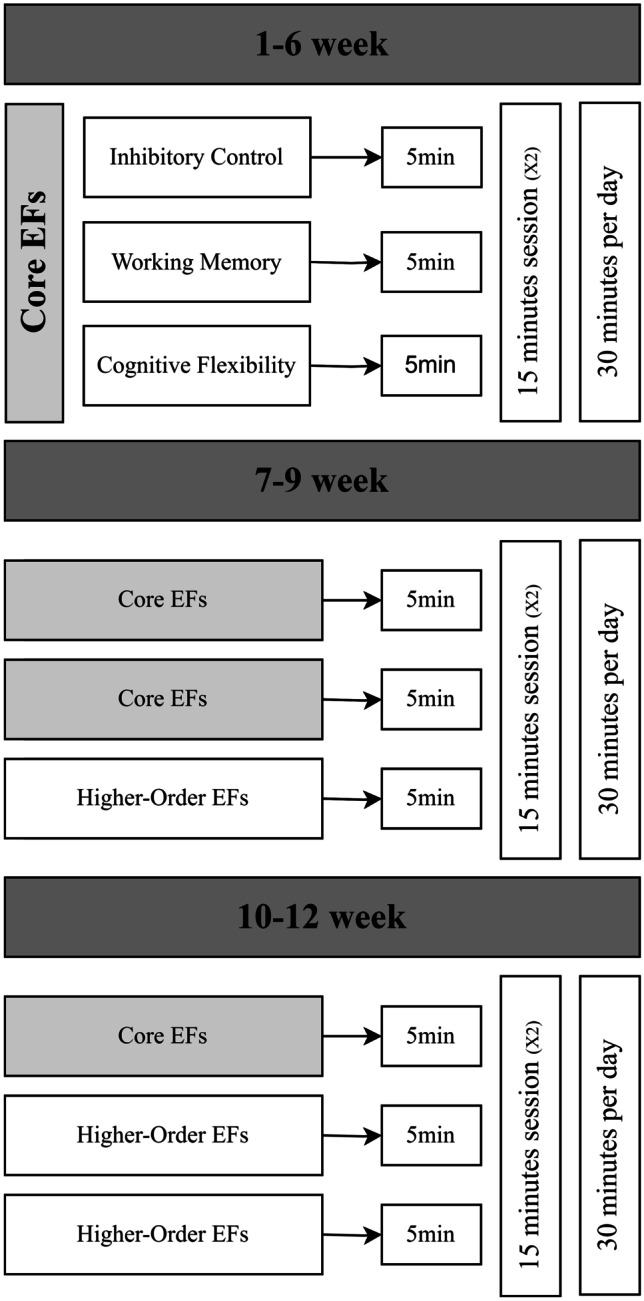
Intervention structure. Abbreviations: EFs, executive functions; X2, twice

Participants were matched in pairs based on age (8–10.5/ 10.6–12 years), sex, Manual Ability Classification System (MACS) level (I–II/III) [32], and intelligence quotient (IQ) score (< 80/ ≥ 80) [33]. Each of the paired participants was then randomized to the intervention (12-week cognitive intervention) or waitlist control (usual care) group. An EF assessment was carried out at three time points: before (T0, baseline), immediately after (T1, postintervention), and 9 months after (T2, follow-up) completing the intervention, as shown in Fig. 1. After finishing the 9-month follow-up assessment, the intervention was offered to the participants in the waitlist control group.

The current study was retrospectively registered on the 19th of July 2019 at ClinicalTrials.gov (NCT04025749). Ethical approval was obtained from the University of Barcelona’s Institutional Ethics Committee Institutional Review Board (IRB 00003099, assurance number: FWA00004225; http://www.ub.edu/recerca/comissiobioetica.htm) and from Sant Joan de Déu—Barcelona Children’s Hospital Ethics Committee (PIC-45–20). The research was conducted in accordance with the Helsinki Declaration. Written informed consent was obtained from all parents or legal guardians of the participants, and oral informed consent was obtained from all participants.

### Participants

Sixty children diagnosed with CP (30 females; mean age 10.3 years, SD 1.6) were recruited from Sant Joan de Déu-Barcelona Children’s Hospital, Hospital Vall d’Hebron, Fundació ASPACE Catalunya; from a previous study of the research group [22]; and through a webpage created for recruitment purposes. The inclusion criteria were (i) children aged 8–12 years; (ii) children presenting with MACS levels I, II, or III; (iii) children who were able to use an intelligible yes/no response system; (iv) children who were able to understand simple instructions as evaluated by the Screening Test of Spanish Grammar [34]; (v) children who were available to participate in the study for a whole year; and (vi) children who had internet access at home. Children were excluded if they had hearing or visual impairments that precluded neuropsychological assessment and cognitive intervention. A total of 60 participants were determined as the required sample size, as described in the study protocol [31].

### Randomization

Participants were randomized using an in-house program written in R by the statistician, which generated the allocation sequence and assigned participants to the intervention or waitlist control group. Once the randomization process was completed, the researcher in charge of the intervention informed the participants’ parents or legal guardians about the group allocation. Participants in the intervention group were informed about the details of the home-based computerized program. The researcher who carried out the assessment remained blinded to the group assignment of each participant throughout the entire study.

### Intervention

NeuronUp (www.neuronup.com) is the computerized cognitive intervention that was used in this project, as detailed in the study protocol [31]. Figure 1 shows the intervention structure. The total proposed dose of the direct intervention was 30 h, distributed over 12 weeks, with a total of 120 sessions (15 min every session), namely, 10 sessions per week (2.5 h per week). During the first 6 weeks, the intervention mainly focused on all three core EFs. The higher-order EF intervention, including some social cognition tasks, was introduced after the sixth week. According to the distribution program, the total intervention dose was 20.6 h for the core EF intervention and 9.4 h for the higher-order EFs/social cognition intervention. The EF intervention started at the basic level of difficulty and was gradually adjusted automatically. Manual adjustment of the sessions was necessary in some cases, such as rescheduling sessions missed due to illness, holiday, or homework.

To mainly ensure that the participants received the full dose, the following adherence strategies were used: (1) *information strategies*: a personalized schedule decorated with pictures related to the children’s interests was delivered, including program instructions, important appointments for neuropsychological assessments, and a special space to record the activities that the children carried out during these 12 weeks. (2) *Flexibility*: each week, each family would elect 5 days across which to complete ten short sessions; (3) *Gaming*: the tasks chosen were those with an appearance similar to videogames. (4) *Motivational monitoring*: during the intervention period, motivational monitoring was carried out by providing personalized immediate messages on platforms such as WhatsApp. Messages were sent weekly to assess motivation and compliance with the previous week’s tasks, which allowed for a minimal standardization of follow-up. After the initial messages, the subsequent messages were always personalized to the children’s preferences and family dynamics. Through these messages, the therapist highlighted positive aspects of the children’s performance to increase motivation and intervention adherence. (5) *Expert diploma*: all participants were informed that after completing the intervention, they would receive a “NeuronUp’s expert diploma.” The same information strategies, motivational monitoring, and expert diploma were applied to the control group.

### Assessment measures

Motor functioning was classified according to the Gross Motor Function Classification System (GMFCS) [35], MACS [32], and Bimanual Fine Motor Function (BFMF) classification [36], and hand function was assessed using the parent-reported Abilhand-Kids scale [37]. Communication skills were classified using the Communication Function Classification System (CFCS) [38] and the Viking Speech Scale (VSS) [39]. Other variables that may have influenced the effect of the intervention were measured by the Bodily Pain and Discomfort Scale of the Child Health Questionnaire (CHQ) [40], Autism Spectrum Screening Questionnaire (ASSQ) [41], Strengths and Difficulties Questionnaire (SDQ) [42], Beach Center Family Quality of Life Scale (fQOL) [43], and Parental Stress Scale (PSS) [44].

Instruments used to measure executive functioning include outcomes of the core EFs (inhibitory control, working memory, and cognitive flexibility) and higher-order EF and their impact on the manifestations of executive functioning in daily life activities. All these instruments were selected considering their reliability and other psychometric properties [31].

#### Inhibitory control

The Digit Span subtest (WISC-V) was used to assess verbal inhibitory control considering forward, backward, and increasing conditions [45]. The Spatial Span subtest (WNV; Wechsler Nonverbal Scale of Ability) was used to assess visual inhibitory control considering forward and backward conditions [46]. Moreover, the Inhibition index (FDT; Five Digit Test) [47] and the Auditory Attention subtest (NEPSY-II) [48] were used.

#### Working memory

Verbal working memory was assessed by using the backward conditions of the Digit Span subtests of the WISC-V [45], while the Spatial Span backward condition of the WNV was selected to assess visual working memory [46].

#### Cognitive flexibility

The Response Set and Word Generation (semantic and initial letter) tests of the NEPSY-II were used to assess cognitive flexibility [48]. Moreover, cognitive flexibility was measured by the Five Digit Test (FDT) [47].

#### Higher-order EFs

The Tower test from the Delis-Kaplan EF System was employed to measure planning skills [49].

#### Manifestations of EF in daily life

To assess behavioral manifestations of executive functioning in everyday life, the parent-proxy version of the Behavior Rating Inventory of Executive Function-2 (BRIEF-2) [51] was used. This scale comprises four indices: the Behavioral Regulation index, Emotional Regulation index, Cognitive Regulation index, and Global Index of EF. In this scale, higher *T* scores indicate poorer performance [51].

### Statistical methods

Per-protocol statistical analyses were performed using IBM SPSS v26 (Statistical Package for the Social Sciences, version 26). Graphical representations were performed with R (version 4.1.0; R Core Team, 2021). Neuropsychological raw data were converted to *z* scores (mean = 0, SD = 1), except for the BRIEF-2 indices, which were converted to *T* scores (mean = 50, SD = 10), based on normative data corrected by age and sex (Table S1). The Shapiro–Wilk test was used to test each variable’s normality. Summary statistics are reported as the mean (standard deviation), median (minimum–maximum), or frequency (percentage) depending on the measurement scale of the variables analyzed. Several physical (pain), mental (autism symptoms and daily difficulties), and environmental (family quality of life and parental stress) variables were considered as potential covariates [40–44]. Correlations between baseline outcomes and these potential covariates were performed (Pearson’s, Spearman’s, or Kendall’s correlation test depending on the measurement scales), applying Bonferroni’s correction (significance level of *p* = 0.01). Only potential covariates that were significantly correlated with the baseline outcomes were included as covariates in our models. To test the effectiveness of the intervention, comparisons between the intervention group and waitlist control group postintervention and at the 9-month follow-up were performed by a series of ANCOVAs (analysis of covariance), with baseline assessments used as covariates in all analyses. The statistical assumptions required for the ANCOVAs, such as the normal distribution of the tested variables, were previously checked. Effect size was assessed by means of the partial eta-squared (${\eta }_{p}^{2}$) index, considering 0–0.05 as small, 0.06–0.13 as medium, and ≥ 0.14 as large effect sizes [52].

Finally, we performed complementary intention-to-treat (ITT) analysis with R (version 4.1.0; R core Team, 2021), which is available as supplementary material (S4 and S5), to assess the potential bias resulting from the withdrawal of 3 participants. For each given outcome, a longitudinal imputation procedure was applied to the data of individuals who underwent the baseline assessment for that outcome (CopyMean-LOCF procedure; [53, 54]). Then, a series of ANCOVAs including the same covariates as the ANCOVAs applied in the per-protocol analysis were performed for each outcome. The imputation procedure carried out in the present study proved to be optimal when there were monotone missing data (for further details, see 54).

## Results

### Participants

Enrolment, allocation, and follow-up are reported according to CONSORT guidelines (Fig. S1) [55]. A total of 140 families were informed about the study and screened for inclusion. Of these 140 families, 53 declined the invitation, and 8 were excluded based on the inclusion and exclusion criteria. Subsequently, 79 children eligible for participation were matched. Prior to the beginning of the study, 16 participants were not included (79% retention rate before the intervention). After randomization, the initial sample included 31 participants in the computerized cognitive intervention group and 32 participants in the waitlist control group. One participant in the intervention group dropped out due to family reasons (97% retention rate after the preintervention assessment). Two participants from the waitlist control group declined to participate due to disagreement with the allocation condition (93% retention rate after the preintervention assessment). The total retention rate was 100% (*n* = 60) during follow-up.

Based on sample size calculation [31] and the withdrawal of 3 participants, the total number of participants that was required to detect changes in the outcome measures was 26 participants in each condition. Thirty participants were included in each group.

The participants’ demographic and clinical characteristics at baseline are presented in Table 1 (mean, standard deviation, interquartile range, number of participants, and percentages). Similarly, the same sample´s descriptive data for potential covariates are presented in Table 2. No significant differences were found between the groups, as shown in Tables 1 and 2. Recruitment took place between November 2017 and December 2020. Postintervention assessments were completed in April 2021, and follow-up assessments were completed in January 2022.

**Table 1:** Descriptive statistics for demographic and clinical data

	Intervention group (*n* = 30)	Control group (*n* = 30)	*T*-Student, Mann–Whitney’s *U*, Chi-squared; *t*/*U*/*χ*^2^	*p*-value
Age, mean ± SD (IQR)	10.3 ± 1.7 (3)	10.0 ± 1.7 (3)	0.50	0.850
Sex, *n* (%)^b^				
Female	15 (50)	15 (50)	< 0.001	1.000
Male	15 (50)	15 (50)		
Gestational age (in weeks), *n* (%)^b^				
< 37 w	14 (46)	20 (66)	0.90	0.824
≥ 37 w	12 (40)	8 (26)		
Unknown	4 (13)	2 (6.7)		
Epilepsy, *n* (%)*				
No epilepsy	24 (80)	18 (60)	2.85	0.091
Active	6 (20)	12 (40)		
Type of CP, *n* (%)^b^				
Spastic	27 (90)	27 (90)	1.20	0.549
Dyskinetic	3 (10)	2 (6.7)		
Unknown	-	1 (3.3)		
Motor distribution, *n* (%)^b^				
Unilateral	24 (80)	24 (80)	4.17	0.243
Bilateral	6 (20)	6 (20)		
GMFCS, *n* (%)^b^				
I	20 (66)	14 (46)	5.78	0.126
II	6 (20)	12 (40)		
III	4 (13)	2 (6.7)		
IV	-	2 (6.7)		
MACS, *n* (%)^b^				
I	11 (36)	14 (46)	0.67	0.715
II	16 (53)	13 (43)		
III	3 (10)	3 (10)		
BFMF, *n* (%)^b^				
I	18 (60)	14 (46)	2.44	0.486
II	8 (26)	12 (40)		
III	3 (10)	4 (6.7)		
IV	1 (3.3)	-		
Abilhand questionnaire, mean ± SD	32.21 ± 7.86	31.03 ± 7.94	0.37	0.713
CFCS, *n* (%)^b^				
I	20 (66)	16 (53)	2.83	0.419
II	9 (30)	10 (33)		
III	1 (3.3)	2 (6.7)		
IV	-	2 (6.7)		
VSS, *n* (%)^b^				
I	26 (86)	18 (60)	5.45	0.065
II	3 (10)	9 (30)		
III	1 (3.3)	3 (10)		
IQ, mean ± SD (IQR)^a^	100.42 ± 15.17 (20)	95.88 ± 9.33 (15)	0.87	0.384

^*^The International League Against Epilepsy criteria were used to determine epilepsy status [65]

*BFMF* Bimanual Fine Motor Function, *CFCS *Communication Function Classification System, *CP* Cerebral palsy, *GMFCS* Gross Motor Function Classification System, *IQ* Intelligence quotient, *IQR* Interquartile range, *MACS* Manual Ability Classification System, *SD* Standard deviation, *VSS* Viking Speech Scale

^a^Mann-Whitney’s *U*

^b^Chi-squared

**Table 2:** Descriptive statistics for potential covariates

	Intervention group	Control group	*T*-Student, Mann–Whitney’s *U*, Chi-squared; *t*/*U*/*χ*^2^	*p-*value
Frequency of pain (CHQ), *n* (%)^b^				
Never	8 (26)	16 (29)	6.47	0.263
A few times	14 (46)	10 (18)		
Often	4 (13)	3 (5.4)		
Unknown	4 (13)	1 (1.8)		
ASSQ, median (IQR)^a^	4.5 (17)	9 (8.0)	354.5	0.692
SDQ, mean ± SD	13.9 ± 6.2	13.7 ± 5.2	−0.1	0.873
fQOL, mean ± SD	3.8 ± 0.7	3.8 ± 0.6	−0.1	0.900
PSS, median (IQR)^a^	22 (14)	25 (10)	329.0	0.154

*ASSQ* Autism Spectrum Screening Questionnaire, *CHQ* Child Health Questionnaire, *fQOL* Beach Center Family Quality of Life Scale, *IQR* interquartile range, *PSS* Parental Stress Scale, *SD* Standard deviation, *SDQ* Strengths and Difficulties Questionnaire

^a^Mann-Whitney’s *U*

^b^Chi-squared

### Intervention

From the 30 h (120 sessions) initially planned for the intervention, a mean of 28.35 h (114 sessions) was completed, with a range between a minimum of 26.30 h (106 sessions) and a maximum of 30 h (120 sessions). The lockdown that took place during the first months of the COVID-19 pandemic in Spain affected the families’ organization, preventing them from carrying out the sessions as planned. Specifically, due to this situation, 3 participants in the intervention group had to extend their training period by five additional weeks, achieving, in the end, the same number of sessions and assessments as the rest of the participants. The mean rate of missed sessions was only 5.0% (a minimum rate of 0.0% and a maximum rate of 13%). Missing data in the analyses due to assessment limitations are specified in the supplementary material.

### Outcomes

Compared to the waitlist control group, the computerized cognitive intervention group showed higher performance immediately after the intervention and follow-up period in some tasks of the three core EFs outcomes, as reported in Tables S2 and S3. Tables S4 and S5 show the ITT analyses for all outcomes included in the study. All results except those for working memory at the 9-month follow-up were the same for the per-protocol and ITT analyses. The covariates used in each analysis and average scores adjusted for covariates in the model (estimated marginal means) are also indicated.

#### Inhibitory control

The intervention group performed better in Spatial Span (*F* = 7.58, *p* = 0.008, ${\eta }_{p}^{2}$= 0.13) than the control group postintervention and in Digit Span (WISC-V) (*F* = 7.85, *p* = 0.007, ${\eta }_{p}^{2}$= 0.12) during follow-up assessments, with a medium effect size.

#### Working memory

The intervention group performed better on the working memory task at postintervention and during follow-up assessments. Specifically, the intervention group performed better on the Spatial Span backward task (WNV; *F* = 8.34, *p* = 0.006, ${\eta }_{p}^{2}$ = 0.14; *F* = 7.55, *p* = 0.008, ${n}_{p}^{2}$ = 0.13) immediately after the intervention (large effect size; *F* = 8.34, *p* = 0.006, ${\eta }_{p}^{2}$ = 0.14) and at the 9-month follow-up (medium effect size; *F* = 7.55, *p* = 0.008, ${n}_{p}^{2}$ = 0.13).

#### Cognitive flexibility

The intervention group performed better on the Response Set task (NEPSY-II) immediately after the intervention (*F* = 4.87, *p* = 0.032, ${\eta }_{p}^{2}$ = 0.09) and at the 9-month follow-up (*F* = 4.19, *p* = 0.046, ${\eta }_{p}^{2}$ = 0.08) assessments, with medium effect sizes.

#### Higher-order EFs

There were no differences between the intervention and waitlist control groups in higher-order EFs (Tower, D-KEFS) and behavioral manifestations of executive functioning in everyday life (BRIEF-2) outcomes immediately and 9 months after the intervention.

### Graphical representations

**Fig. 2:**
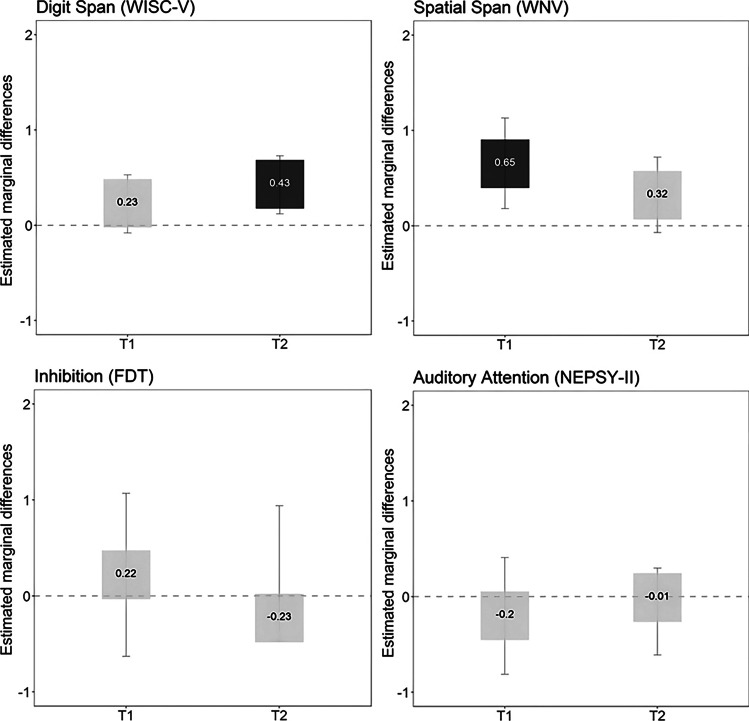
Graphical representation of differences between intervention and waitlist groups in inhibitory control. Notes: dark gray box (significant differences between the intervention and waitlist group); light gray (no significant differences). Estimated marginal differences (estimated marginal mean of the intervention group − estimated marginal mean of the waitlist control group) above zero indicate that the intervention group has better performance than the waitlist group. Whiskers correspond to the 95% CIs for the marginal differences. Abbreviations: T1, postintervention; T2, 9-month follow-up after the intervention; FDT, Five Digit Test; NEPSY-II, a Developmental Neuropsychological Assessment, Second Edition; WISC-V, Wechsler Intelligence Scale for Children, Fifth Edition; WNV, Wechsler Nonverbal Scale of Ability

**Fig. 3:**
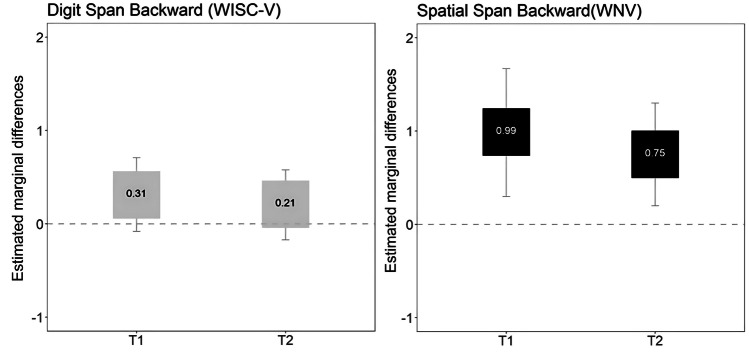
Graphical representation of differences between intervention and waitlist groups in working memory. Notes: dark gray box (significant differences between the intervention and waitlist group); light gray (no significant differences). Estimated marginal differences (estimated marginal mean of the intervention group − estimated marginal mean of the waitlist control group) above zero indicate that the intervention group has better performance than the waitlist group. Whiskers correspond to the 95% CIs for the marginal differences. Abbreviations: T1, postintervention; T2, 9-month follow-up after intervention; WISC-V, Wechsler Intelligence Scale for Children, Fifth Edition; WNV, Wechsler Nonverbal Scale of Ability

**Fig. 4:**
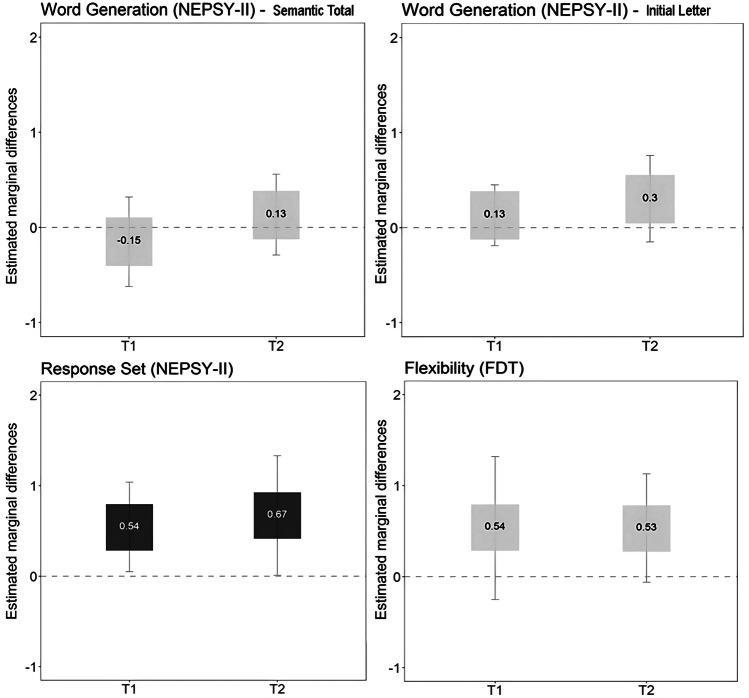
Graphical representation of differences between intervention and waitlist groups in cognitive flexibility graphical representation. Notes: dark gray box (significant differences between the intervention and waitlist group); light gray (no significant differences). Estimated marginal differences (estimated marginal mean of the intervention group − estimated marginal mean of the waitlist control group) above zero indicate that the intervention group has better performance than the waitlist group. Box sizes represent the magnitude of the estimated differences (i.e., areas are proportional to the corresponding estimated effect), whereas wWhiskers correspond to the 95% CIs for the marginal differences. Abbreviations: T1, postintervention; T2, 9-month follow-up after intervention; FDT, Five Digit Test; NEPSY-II, a Developmental Neuropsychological Assessment, Second Edition

The graphical representations of the results are presented in Figs. 2, 3, and 4 and Figs. S2 and S3. Boxes represent the estimated marginal differences (differences between the groups’ estimated marginal means in Tables S2 and S3) between the intervention and waitlist control groups immediately after the intervention (T1) and at the 9-month follow-up (T2). The intervention group showed better performance on the majority of the cognitive domains than the waitlist control group (positive differences shown in Figs. 2, 3, and 4 and Fig. S2; negative differences shown in Fig. S3), although not all reached significance (dark gray boxes). The graphical representation also shows two different patterns in some measures. First, there was an ascending pattern in which changes were higher at the 9-month follow-up than immediately postintervention, as seen, for example, in the Digit Span (WISC-V) plot (Fig. 2). Second, there was a descending pattern in which the highest differences were in the postintervention assessment compared to the 9-month follow-up assessment, as seen, for example, in the Spatial Span (WNV) plot (Fig. 2).

## Discussion

The main findings of this study suggest that a computerized intensive and progressively challenging EF home intervention improved core EFs performance (inhibitory control, working memory, and cognitive flexibility) in children with CP. Core EF differences between the groups in some tasks were also demonstrated at the 9-month follow-up after completing the intervention. To our knowledge, this is the first study to demonstrate that a 30-h cognitive intervention improved performance in some tasks covering all core EFs.

This positive effect in the core EFs may translate to large and significant improvements in behavior, attention, thoughts, and/or emotional control [56]. Specifically, improvements in inhibitory control meant that children’s ability to focus on what they chose increased, instead of focusing on making better decisions about what was more appropriate or needed, by suppressing their attention to other stimuli [11]. These improvements align with previous RCTs with a low risk of bias that showed significant improvements in inhibitory control with multimodal and cognitive interventions immediately after the interventions [28]. Long-term effects, however, were not explored in these studies. Working memory changes, reported in the present study, implied that children’s capacity to hold information in mind for a short time and mentally process it increased [11]. Consistent with our results, Di Lieto et al. [27] found that children with CP presented significant improvements in working memory immediately after receiving a 5-week computerized cognitive intervention but not at follow-up [21]. Finally, cognitive flexibility improvements suggested that the intervention increased the children’s ability to be more flexible between different tasks. This flexibility may allow children to adjust to changing demands or priorities, to admit what is wrong, and to take advantage of sudden unexpected opportunities [11]. Previous studies have not shown beneficial effects on cognitive flexibility after multimodal or cognitive interventions [26, 27, 56]. Although Mak et al. [26] in 2018 did not find improvements in cognitive flexibility immediately after the intervention, delayed effects were found 6 months later [29].

Improvements in higher-order EFs have only been found in reasoning using a reality-based rehabilitation program for children with CP [30]. In the present study, we did not find significant differences in higher-order EFs related to planning. Previous RCTs with a low risk of bias also showed no improvements using the same planning measure (Tower test) as that used in the present study [56]. Piovesana et al. [56] discussed that their negative results might be because the cognitive challenges of their multimodal intervention were not focused on EFs. In the present study, the intervention specifically targeted EFs. We therefore concluded that the lack of beneficial effects on higher-order EF outcomes could be due to insufficient training time dedicated to higher-order EF tasks. Specifically, from the 30-h total dose in the present study, only 33% of the dose corresponded to tasks targeting higher-order EFs. Future studies should explore whether increasing the dose of higher-order EF training results in significant improvements in this domain.

Identifying the optimal dose is a key factor to guarantee maximum adherence to treatment. Such an optimal dose allowing positive results in core EFs is highly variable in previous studies. For example, Ahn et al. [19] found that the optimal intervention dose was 21.3 h, reported as a mean. In the study by Di Lieto et al. [27], the intervention dose ranged from 8 to 18 h. Finally, Mak et al. [26] reported 12.5 h as the mean intervention dose. In the present study, we allowed flexibility for reaching the total dose (each week, the participants could elect the 5 days across which to complete the ten sessions, and several motivational strategies were used to improve participant and family engagement). These aspects allowed a small variability in the total dose achieved (26 to 30 h), which represents an attrition rate of only 5%.

Once the optimal dose has been identified, computerized interventions such as the one in this study may also be key to achieving it. Computerized cognitive interventions can be beneficial for children with various conditions, including CP, attention-deficit/hyperactivity disorder (ADHD), autism spectrum disorder (ASD), and learning difficulties [57, 58]. These interventions improved cognitive skills, such as working memory and flexibility skills, in children with ADHD or ASD [59, 60]. A systematic review of children with and without neuropsychological disorders also stated that this type of intervention is effective in improving cognitive skills [57]. Additionally, creating EF intervention opportunities outside of the rehabilitation center setting could provide more parental agency in the rehabilitation process and a more collaborative and supportive approach to an intervention in the natural environment, leading to greater compliance with programs, reducing stress levels, and improving outcomes.

Our results did not prove positive effects on manifestations of executive functioning in daily life, assessed with a rating scale that measures core and higher-order EFs. The results of this study reinforce the idea that performance-based tests and rating scales assess different EF aspects [9, 20]. Both instruments are complementary and should be used for assessing EFs. In this way, this could help to characterize the impairment and consequently prove the right support for patients and caregivers. Overall, our results are consistent with previous studies in other child populations related to EFs, such as populations with ASD, ADHD, or learning difficulties, in which transfer effects on manifestations of EF in daily life have not been reported [9, 20, 21]. Thus, further research is needed to clarify intervention characteristics and the assessment tools used to check intervention effects. These studies may also propose interventions that include face-to-face interventions and specific professional advice that allow the transfer of changes to manifestations of EF in daily life.

A limitation of the present study is that it did not include children across all MACS levels. Only participants at levels I–III were included to homogenize the characteristics of the sample and the effective time of the cognitive intervention among participants. Moreover, adaptations in the cognitive assessment were applied for one participant with vision impairment. In this participant, a computerized, instead of paper, version of the FDT was used. Additionally, we did not include an active control group because almost all cognitive tasks imply some level of EF. Other factors might be of interest to consider in cognitive intervention effectiveness, such as nutritional status [61, 62] or sleep disorders [63]. In addition, some families needed three more weeks (15 weeks instead of 12) to reach the total dose due to the COVID-19 pandemic. Finally, the pandemic might also have influenced, to some degree, children’s responses to treatment due to the potential decrease in their general health as a result of the disruption in health and rehabilitation services [64].

## Conclusions

Our results indicate that a home-based computerized EF intervention, together with motivational monitoring strategies that enhance adherence, can improve the core EFs of children with CP for at least 9 months postintervention. This intervention could be complementary to conventional face-to-face therapies to intensively stimulate cognitive functioning in children with CP. Further research is needed to identify strategies that allow the improvements to be transferred to everyday life and to test this intervention across the full spectrum of severity that people with CP can present.

### Supplementary Information

Below is the link to the electronic supplementary material.

Supplementary file1 (DOCX 505 KB)

## Data Availability

Online resources are available. All data relevant to the study are included in the article or uploaded as supplementary information. Original data are available from the corresponding author upon request.
