# Supplementary material for: A randomized controlled trial of a home-based computerized executive function intervention for children with cerebral palsy
Source: Eur J Pediatr. 2023 Jul 18;182(10):4351–63. doi: 10.1007/s00431-023-05072-3 (PMC10587273; doi:10.1007/s00431-023-05072-3)
Supplement: Supplementary file 1 — Supplementary file1 (DOCX 505 KB) [file 431_2023_5072_MOESM1_ESM.docx]

**Title:** A randomized controlled trial of a home-based computerized executive function intervention for children with cerebral palsy - Supplementary material

**Journal:** European Journal of Pediatrics.

**Authors:** García-Galant M_1,2,3_, Blasco M_1,2,3_, Laporta-Hoyos O_1,2,3_, Berenguer-González A_1_, Moral-Salicrú P_1_, Ballester-Plané J_1,2,3_, Caldú X_1,2,3_, Miralbell J_1,2,3_, Alonso X_4_, Medina-Cantillo J_5_, Povedano-Bulló E_5_, Leiva D_6_, Boyd R.N_7_, Pueyo R_1,2,3_*

1. Departament de Psicologia Clínica i Psicobiologia, Universitat de Barcelona, Passeig de la Vall d’Hebron, 171, 08035, Barcelona, Spain
2. Institut de Neurociències, Universitat de Barcelona, Passeig de la Vall d’Hebron, 171, 08035, Barcelona, Spain
3. Institut de Recerca Sant Joan de Déu, Santa Rosa 39-57, 08950, Esplugues de Llobregat, Barcelona, Spain
4. Servei de Neurologia, Hospital Sant Joan de Déu, Passeig de Sant Joan de Déu, 2, 08950, Esplugues de Llobregat, Barcelona, Spain
5. Servei de Rehabilitació i Medicina Física, Passeig de Sant Joan de Déu, 2, 08950, Esplugues de Llobregat, Barcelona, Spain
6. Departament de Psicologia Social i Psicologia Quantitativa, Universitat de Barcelona, Passeig de la Vall d’Hebron, 171, 08035, Barcelona, Spain.
7. Queensland Cerebral Palsy and Rehabilitation Research Centre, Faculty of Medicine, The University of Queensland, 62 Graham St., Brisbane, 4101, Queensland, Australia

* Corresponding author at: Passeig de la Vall d’Hebron, 171, 08035, Barcelona, Spain

E-mail address: [rpueyo@ub.edu](mailto:rpueyo@ub.edu)

ORCID: [0000-0002-8230-8409](http://orcid.org/0000-0002-8230-8409)

Fax number: [+34] 93 402 1584

**Supplementary Material**

| **Table S1**  Mean (SD) and median (minimum/maximum) Z scores on the neuropsychological assessment tasks of the intervention and waitlist groups at baseline. | | | | | | | | | | | | | | | | |
| --- | --- | --- | --- | --- | --- | --- | --- | --- | --- | --- | --- | --- | --- | --- | --- | --- |
|  | | |  |  | | EF Intervention group | | | | | Waitlist group | | | | | |
|  |  |  | Baseline | | | Post-intervention | | | Follow-Up | | Baseline | | Post-intervention | | Follow-Up | |
| Executive Functions | | | n | Mean*  (SD)  Median  (range) | | n | Mean*  (SD)  Median  (range) | | n | Mean*  (SD)  Median  (range) | n | Mean*  (SD)  Median  (range) | n | Mean*  (SD)  Median  (range) | n | Mean*  (SD)  Median  (range) |
|  | Inhibitory Control | | | | | | | | | | | | | | | |
|  | Digit Span  (WISC-V) | | 30 | -0.67  (-3.00/0.67) | | 30 | -0.33  (-3.00/1.00) | | 30 | -0.67  (-0.00/1.67) | 29^a^ | -1.44*  (0.17) | 29^c^ | -1.44*  (0.17) | 29^c^ | -1.6*  (0.17) |
|  | Spatial Span (WNV) | | 30 | -0.91*  (0.22) | | 30 | -0.53*  (0.28) | | 30 | -0,64*  (0.23) | 29^c^ | -1.52*  (0.20) | 30 | -1.63*  (0.20) | 30 | -1.37* (0.18) |
|  | Inhibition (FDT) | | 29^c^ | -1.22  (-2.33/2.33) | | 30 | -0.52  (-2.33/2.33) | | 29^c^ | -0.84  (-2.33/2.33) | 26^c^ | -1.75  (-2.33/2.33) | 27^c^ | -0.84  (-2.33/2.33) | 27^c^ | -1.28  (-2.33/2.33) |
|  | Auditory Attention (NEPSY-II) | | 30 | 0.50  (-3.00/1.33) | | 29^c^ | 0.33  (-3.00/1.33) | | 30 | 0.00  (-2.67/1.50) | 30 | -1.33  (-3.00/1.00) | 30 | -0.97  (-3.00/1.50) | 29^c^ | -0.33  (-3.00/1.50) |
|  | Working Memory | | | | | | | | | | | | | | | |
|  | Digit Span Backward  (WISC-V) | | 30 | -0.59  (-4.4/0.91) | | 30 | -0.55*  (0.20) | | 30 | -0.65*  (0.84) | 29^a^ | -1.09*  (0.25) | 29^c^ | -0.90  (-4.40/1.10) | 29^c^ | -1.33  (-3.00/0.33) |
|  | Spatial Span Backward* (WNV) | | 30 | -0.64*  (0.27) | | 30 | -1.57*  (0.30) | | 30 | -0.55  (-5.10/1.25) | 29^a^ | -0.90  (-5.10/1.00) | 30 | -1.28*  (0.23) | 30 | -1.30*  (0.25) |
|  | Cognitive Flexibility | | | | | | | | | | | | | | | |
|  | Word Generation (NEPSY-II) | | | | | | | | | | | | | | | |
|  | Semantic Total | | 30 | -0.56*  (0.19) | | 30 | -0.71*  (0.21) | | 30 | -0.49*  (0.19) | 28^a^ | -0.89*  (0.26) | 28^a^ | -0.79*  (0.26) | 28^a^ | -1.16  (-3.00/1.33) |
|  | Initial Letter | | 30 | -1.06*  (0.16) | | 30 | -3.74*  (0.14) | | 30 | -0.63*  (0.21) | 28^a^ | -1.34*  (0.15) | 27^ac^ | -4.30  (-5.00/-2.00) | 28^a^ | -1.33  (-2.6/1.67) |
|  | Response Set  (NEPSY-II) | | 30 | -0.58*  (0.25) | | 29^c^ | -0.33  (-3.00/1.67) | | 30 | 0.16  (-3.00/1.50) | 30 | -1.50  (-3.00/1.00) | 30 | -1.67  (-3.00/1.00) | 28^cd^ | -1.16  (-3.00/1.50) |
|  | Flexibility  (FDT) | | 29^c^ | -0.52  (-2.33/2.33) | | 30 | -0.45  (-2.33/2.33) | | 29^c^ | <0.01*  (0.17) | 26^c^ | -2.05  (-2.33/2.33) | 27^c^ | -2.32  (-2.33/2.33) | 28^c^ | <0.01  (0.20) |
|  | Higher-Order Executive Functions | | | | | | | | | | | | | | | |
|  | Tower  (D-KEFS) | | 30 | -0.41*  (0.20) | | 30 | 0.33  (-3.00/2.33) | | 30 | 0.33  (-3.00/2.33) | 29^b^ | -1.00  (-3.00/0.33) | 28^c^ | -0.67  (-2.6/1.00) | 28^bc^ | -0.59*  (0.24) |
|  | Parental Executive function Questionnaires | | | | | | | | | | | | | | | |
|  | BRIEF-2 (T-scores) | |  | |  | | |  | | | | | | | | |
|  |  | Behavioural Regulation Index | 30 | 62.17*  (2.28) | | 27^e^ | 56.00*  (41.00/92.00) | | 26^e^ | 59.58*  (2.69) | 29^e^ | 61.14*  (2.01) | 29^e^ | 61.03*  (1.88) | 27^e^ | 62.00  (50.00/83.00) |
|  |  | Emotional  Regulation Index | 30 | 65.83*  (2.20) | | 27^e^ | 65.26*  (2.76) | | 26^e^ | 67.19*  (2.88) | 29e | 61.69*  (1.97) | 29^e^ | 61.83*  (2.08) | 27^e^ | 61.78  (2.21) |
|  |  | Cognitive Regulation Index | 29^e^ | 63.97*  (2.22) | | 27^e^ | 62.96*  (2.76) | | 26^e^ | 63.57  (2.65) | 28^e^ | 63.82*  (1.8) | 28^e^ | 65.07*  (2.01) | 26^e^ | 64.03*  (1.99) |
|  | Global Index of EF | | 29^e^ | 66.31*  (2.24) | | 27^e^ | 64.56*  (2.90) | | 26^e^ | 65.54  (2.83) | 28^e^ | 64.54*  (1.91) | 28^e^ | 65.39*  (2.04) | 26^e^ | 65.04  (2.00) |

Notes: Reasons for missing data: a = very slow communication system that precluded the use of an appropriate response system for the test used; b = severe motor impairment that made it difficult to complete the task; c = being not able to complete the test due to fatigue; d = lockdown precluded assessment, e = not answered by the parents.

Abbreviations: BRIEF-2 = Behavior Rating Inventory of Executive Function-Second Edition;
EF = Executive function; D-KEFS = Delis–Kaplan Executive Function System; FDT = Five Digit Test; NEPSY-II = A Developmental Neuropsychological Assessment, Second Edition;
SD = Standard deviation; WISC-V = Wechsler Intelligence Scale for Children-Fifth Edition; WNV = Wechsler Nonverbal Scale of Ability.

| **Table S2**  Analysis of covariance comparing intervention and waitlist groups on EF postintervention outcomes | | | | | | | | | | |  |  |  |
| --- | --- | --- | --- | --- | --- | --- | --- | --- | --- | --- | --- | --- | --- |
| Outcomes | | | Intervention Group | | Control Group | | ANCOVA  Post-intervention | | | | | |  |
|  |  |  | Estimated marginal mean  (SD) | | Estimated marginal mean  (SD) | | F | | *p* | | $n_{p}^{2}$ | |  |
| Executive Functions | | |  | |  | |  | |  | |  | |  |
| Inhibitory Control | | | | | | | | | | | | |  |
|  | Digit Span (WISC-V) | | | -0.87  (0.10) | | -1.10 (0.10) | | 2.13 | | 0.150 | | 0.03 | |
|  | Spatial Span (WNV) ^ac^ | | | -0.70  (0.16) | | -1.36  (0.16) | | 7.58 | | 0.008 | | 0.13 | |
|  | Inhibition (FDT) ^b^ | | | -0.26  (0.28) | | -0.48  (0.30) | | 0.26 | | 0.608 | | 0.01 | |
|  | Auditory Attention (NEPSY-II) ^c^ | | | -0.73  (0.20) | | -0.53 (0.19) | | 0.43 | | 0.514 | | 0.01 | |
| Working Memory | | | | | | | | | | | | |  |
|  | Digit Span Backward  (WISC-V) ^c^ | | | -0.51 (0.13) | | -0.82 (0.14) | | 2.49 | | 0.120 | | 0.04 | |
|  | Spatial Span Backward (WNV) ^ab^ | | | -0.17 (0.23) | | -0.15 (0.23) | | 8.34 | | 0.006 | | 0.14 | |
| Cognitive Flexibility | | |  | |  | |  | |  | |  | |  |
|  | Word Generation (NEPSY-II) | | |  | |  | |  | |  | |  | |
|  | Semantic Total ^c^ | | | -0.80 (0.16) | | -0.65 (0.16) | | 0.40 | | 0.526 | | <0.01 | |
|  | Initial Letter | | | -3.82 (0.10) | | -3.95 (0.11) | | 0.68 | | 0.412 | | 0.01 | |
|  | Response Set (NEPSY-II) ^ac^ | | | -0.41 (0.16) | | -0.95 (0.16) | | 4.87 | | 0.032 | | 0.09 | |
|  | Flexibility (FDT) | | | -0.49  (0.26) | | -1.03  (0.28) | | 1.86 | | 0.178 | | 0.03 | |
| Higher-Order Executive Functions | | | | | | | | | | | | |  |
|  | Tower (D-KEFS) | | | -0.11 (0.18) | | -0.39 (0.18) | | 1.09 | | 0.300 | | 0.02 | |
| Manifestations of EF in daily life | | | | | | | | | | | | |  |
|  | BRIEF-2 (T-scores) | | |  | |  | |  | |  | |  | |
|  |  | Behavioural Regulation Index^abc^ | | 57.63 (1.24) | | 60.76 (1.24) | | 3.03 | | 0.088 | | 0.06 | |
|  |  | Emotional Regulation Index^ac^ | | 63.11 (1.55) | | 63.04 (1.55) | | <0.01 | | 0.978 | | <0.01 | |
|  |  | Cognitive Regulation Index^ac^ | | 62.58 (1.10) | | 64.03 (1.10) | | 0.85 | | 0.360 | | 0.02 | |
|  | Global Index of Executive Function ^abc^ | | | 63.44 (1.10) | | 65.30 (1.10) | | 1.37 | | 0.248 | | 0.03 | |

Abbreviations: BRIEF-2 = Behavior Rating Inventory of Executive Function-Second Edition;
EF = Executive function; D-KEFS = Delis–Kaplan Executive Function System; FDT = Five Digit Test; NEPSY-II = A Developmental Neuropsychological Assessment, Second Edition;
SD = Standard deviation; WISC-V = Wechsler Intelligence Scale for Children-Fifth Edition; WNV = Wechsler Nonverbal Scale of Ability. Covariates: ^a^ ASSQ = Autism Spectrum Screening Questionnaire; ^b^PSS = Parental Stress Scale; ^c^ SDQ = Strengths and Difficulties Questionnaire.

**Table S3**

Analysis of covariance comparing intervention and waitlist groups on EF follow-up outcomes

| Outcomes | | | Intervention Group | Waitlist Group | ANCOVA  Follow-up | | |
| --- | --- | --- | --- | --- | --- | --- | --- |
|  |  |  | Estimated marginal mean  (SD) | Estimated marginal mean  (SD) | F | *p* | $n_{p}^{2}$ |
| Executive Functions | | |  |  |  |  |  |
| Inhibitory Control | | | | | | | |
|  | Digit Span (WISC-V) | | -0.88  (0.10) | -1.31 (0.10) | 7.85 | 0.007 | 0.12 |
|  | Spatial Span (WNV) ^ac^ | | -0.77  (0.13) | -1.10  (0.13) | 2.70 | 0.106 | 0.05 |
|  | Inhibition (FDT) ^b^ | | -0.74  (0.23) | -0.97 (0.25) | 0.44 | 0.510 | <0.01 |
|  | Auditory Attention (NEPSY-II) ^c^ | | -0.39  (0.20) | -0.40 (0.20) | <0.01 | 0.976 | <0.01 |
| Working Memory | | | | | | | |
|  | Digit Span Backward (WISC-V) ^c^ | | -0.88 (0.13) | -1.09 (0.13) | 1.19 | 0.280 | 0.02 |
|  | Spatial Span Backward (WNV)^ab^ | | -0.41 (0.18) | -1.16 (0.18) | 7.55 | 0.008 | 0.13 |
| Cognitive Flexibility | | |  |  |  |  |  |
|  | Word Generation (NEPSY-II) | |  |  |  |  |  |
|  | Semantic Total^c^ | | -0.58 (0.14) | -0.71 (0.15) | 0.39 | 0.532 | <0.01 |
|  | Initial Letter | | -0.75 (0.15) | -1.05 (0.16) | 1.78 | 0.187 | 0.03 |
|  | Response Set (NEPSY-II) ^ac^ | | -0.07 (0.21) | -0.74 (0.23) | 4.19 | 0.046 | 0.08 |
|  | Flexibility (FDT) | | -0.83  (0.20) | -1.36  (0.21) | 3.19 | 0.080 | 0.06 |
| Higher-Order Executive Functions | | | | | | | |
|  | Tower (D-KEFS) | | -0.03 (0.14) | -0.26 (0.14) | 1.27 | 0.265 | 0.02 |
| Manifestations of EF in daily life | | | | | | | |
|  | BRIEF-2 (T-scores) | |  |  |  |  |  |
|  |  | Behavioural Regulation Index ^abc^ | 58.83 (1.27) | 62.39 (1.24) | 3.82 | 0.057 | 0.07 |
|  |  | Emotional Regulation Index ^ac^ | 64.93 (1.90) | 63.98 (1.86) | 0.12 | 0.727 | <0.01 |
|  |  | Cognitive Regulation Index ^ac^ | 63.43 (1.48) | 63.68 (1.48) | 0.01 | 0.906 | <0.01 |
|  | Global Index of Executive Function ^abc^ | | 65.16 (1.44) | 64.95 (1.44) | 0.01 | 0.921 | <0.01 |

Abbreviations: BRIEF-2 = Behavior Rating Inventory of Executive Function-Second Edition;
EF = Executive function; D-KEFS = Delis–Kaplan Executive Function System; FDT = Five Digit Test; NEPSY-II = A Developmental Neuropsychological Assessment, Second Edition;
SD = Standard deviation; WISC-V = Wechsler Intelligence Scale for Children-Fifth Edition; WNV = Wechsler Nonverbal Scale of Ability. Covariates: ^a^ ASSQ = Autism Spectrum Screening Questionnaire; ^b^PSS = Parental Stress Scale; ^c^ SDQ = Strengths and Difficulties Questionnaire.

| **Table S4**  Intention to treat analysis of covariance comparing intervention and waitlist groups on EF postintervention outcomes | | | | | | | | | | |  |  |  |
| --- | --- | --- | --- | --- | --- | --- | --- | --- | --- | --- | --- | --- | --- |
| Outcomes | | | Intervention Group | | Control Group | | ANCOVA  Post-intervention | | | | | |  |
|  |  |  | Estimated marginal mean  (SD) | | Estimated marginal mean  (SD) | | F | | *p* | | $n_{p}^{2}$ | |  |
| Executive Functions | | |  | |  | |  | |  | |  | |  |
| Inhibitory Control | | | | | | | | | | | | |  |
|  | Digit Span (WISC-V) | | | -0.95  (0.10) | | -1.16 (0.10) | | 2.30 | | 0.135 | | <0.01 | |
|  | Spatial Span (WNV) ^ab^ | | | -0.72  (0.16) | | -1.40  (0.16) | | 6.00 | | 0.018 | | 0.14 | |
|  | Inhibition (FDT)^c^ | | | -0.36  (0.25) | | -0.58  (0.26) | | 0.38 | | 0.540 | | <0.01 | |
|  | Auditory Attention (NEPSY-II)^b^ | | | -0.74  (0.19) | | -0.53 (0.19) | | 0.63 | | 0.431 | | <0.01 | |
| Working Memory | | | | | | | | | | | | |  |
|  | Digit Span Backward  (WISC-V) ^b^ | | | -0.59 (0.15) | | -0.91 (0.15) | | 2.40 | | 0.127 | | <0.01 | |
|  | Spatial Span Backward (WNV) ^ac^ | | | -0.17 (0.23) | | -0.16 (0.24) | | 7.52 | | 0.008 | | 0.03 | |
| Cognitive Flexibility | | |  | |  | |  | |  | |  | |  |
|  | Word Generation (NEPSY-II) | | |  | |  | |  | |  | |  | |
|  | Semantic Total ^b^ | | | -0.82 (0.16) | | -0.67 (0.16) | | 0.51 | | 0.477 | | <0.01 | |
|  | Initial Letter | | | -0.8 (0.16) | | -1.04 (0.16) | | 1.15 | | 0.287 | | <0.01 | |
|  | Response Set (NEPSY-II) ^ab^ | | | -0.47 (0.16) | | -1.00 (0.16) | | 6.49 | | 0.014 | | 0.12 | |
|  | Flexibility (FDT) | | | -0.62  (0.25) | | -1.17  (0.24) | | 2.43 | | 0.124 | | <0.01 | |
| Higher-Order Executive Functions | | | | | | | | | | | | |  |
|  | Tower (D-KEFS) | | | -0.16 (0.17) | | -0.41 (0.17) | | 1.04 | | 0.311 | | <0.01 | |
| Manifestations of EF in daily life | | | | | | | | | | | | |  |
|  | BRIEF-2 (T-scores) | | |  | |  | |  | |  | |  | |
|  |  | Behavioural Regulation Index^abc^ | | 58.46 (1.87) | | 60.27 (1.91) | | 0.49 | | 0.488 | | <0.01 | |
|  |  | Emotional Regulation Index^ab^ | | 63.38 (1.39) | | 63.54 (1.42) | | 0.05 | | 0.824 | | <0.01 | |
|  |  | Cognitive Regulation Index^ab^ | | 62.52 (0.97) | | 64.06 (1.00) | | 1.06 | | 0.308 | | 0.02 | |
|  | Global Index of Executive Function ^abc^ | | | 66.81 (2.48) | | 63.58 (2.53) | | 0.42 | | 0.518 | | <0.01 | |

Abbreviations: BRIEF-2 = Behavior Rating Inventory of Executive Function-Second Edition;
EF = Executive function; D-KEFS = Delis–Kaplan Executive Function System; FDT = Five Digit Test; NEPSY-II = A Developmental Neuropsychological Assessment, Second Edition;
SD = Standard deviation; WISC-V = Wechsler Intelligence Scale for Children-Fifth Edition; WNV = Wechsler Nonverbal Scale of Ability. Covariates: ^a^ ASSQ = Autism Spectrum Screening Questionnaire; ^b^PSS = Parental Stress Scale; ^c^ SDQ = Strengths and Difficulties Questionnaire.

| **Table S5**  Intention to treat analysis of covariance comparing intervention and waitlist groups on EF follow-up outcomes | | | | | | | | | | |  |  |  |
| --- | --- | --- | --- | --- | --- | --- | --- | --- | --- | --- | --- | --- | --- |
| Outcomes | | | Intervention Group | | Control Group | | ANCOVA  Follow-Up | | | | | |  |
|  |  |  | Estimated marginal mean  (SD) | | Estimated marginal mean  (SD) | | F | | *p* | | $n_{p}^{2}$ | |  |
| Executive Functions | | |  | |  | |  | |  | |  | |  |
| Inhibitory Control | | | | | | | | | | | | |  |
|  | Digit Span (WISC-V) | | | -1.06  (0.09) | | -1.43 (0.09) | | 8.38 | | 0.005 | | <0.01 | |
|  | Spatial Span (WNV) ^ab^ | | | -0.80  (0.13) | | -1.34  (0.13) | | 1.25 | | 0.269 | | 0.14 | |
|  | Inhibition (FDT) c | | | -0.80  (0.22) | | -1.06  (0.23) | | 0.99 | | 0.323 | | <0.01 | |
|  | Auditory Attention (NEPSY-II)^b^ | | | -0.48  (0.20) | | -0.38 (0.20) | | 0.08 | | 0.772 | | <0.01 | |
| Working Memory | | | | | | | | | | | | |  |
|  | Digit Span Backward  (WISC-V) ^c^ | | | -0.97 (0.14) | | -1.12 (0.13) | | 0.69 | | 0.408 | | <0.01 | |
|  | Spatial Span Backward (WNV) ^ac^ | | | -0.49 (0.23) | | -1.15 (0.23) | | 8.52 | | 0.067 | | 0.03 | |
| Cognitive Flexibility | | |  | |  | |  | |  | |  | |  |
|  | Word Generation (NEPSY-II) | | |  | |  | |  | |  | |  | |
|  | Semantic Total ^b^ | | | -0.6  (0.14) | | -0.74 (0.14) | | 0.33 | | 0.566 | | <0.01 | |
|  | Initial Letter | | | -0.74 (0.15) | | -1.03 (0.15) | | 1.84 | | 0.180 | | <0.01 | |
|  | Response Set (NEPSY-II) ^ab^ | | | -0.14 (0.21) | | -0.82 (0.21) | | 6.04 | | 0.017 | | 0.12 | |
|  | Flexibility (FDT) | | | -0.98  (0.19) | | -1.49  (0.18) | | 3.63 | | 0.062 | | <0.01 | |
| Higher-Order Executive Functions | | | | | | | | | | | | |  |
|  | Tower (D-KEFS) | | | -0.05 (0.14) | | -0.32 (0.14) | | 1.93 | | 0.170 | | <0.01 | |
| Manifestations of EF in daily life | | | | | | | | | | | | |  |
|  | BRIEF-2 (T-scores) | | |  | |  | |  | |  | |  | |
|  |  | Behavioural Regulation Index^abc^ | | 58.55 (1.88) | | 62.20 (1.88) | | 2.51 | | 0.119 | | 0.05 | |
|  |  | Emotional Regulation Index^ab^ | | 64.44 (1.75) | | 64.00 (1.78) | | <0.01 | | 0.945 | | <0.01 | |
|  |  | Cognitive Regulation Index^ab^ | | 63.12 (1.37) | | 63.81 (1.42) | | 0.03 | | 0.856 | | <0.01 | |
|  | Global Index of Executive Function ^abc^ | | | 64.31 (1.33) | | 64.66 (1.36) | | 0.44 | | 0.509 | | <0.01 | |

Abbreviations: BRIEF-2 = Behavior Rating Inventory of Executive Function-Second Edition;
EF = Executive function; D-KEFS = Delis–Kaplan Executive Function System; FDT = Five Digit Test; NEPSY-II = A Developmental Neuropsychological Assessment, Second Edition;
SD = Standard deviation; WISC-V = Wechsler Intelligence Scale for Children-Fifth Edition; WNV = Wechsler Nonverbal Scale of Ability. Covariates: ^a^ ASSQ = Autism Spectrum Screening Questionnaire; ^b^PSS = Parental Stress Scale; ^c^ SDQ = Strengths and Difficulties Questionnaire.


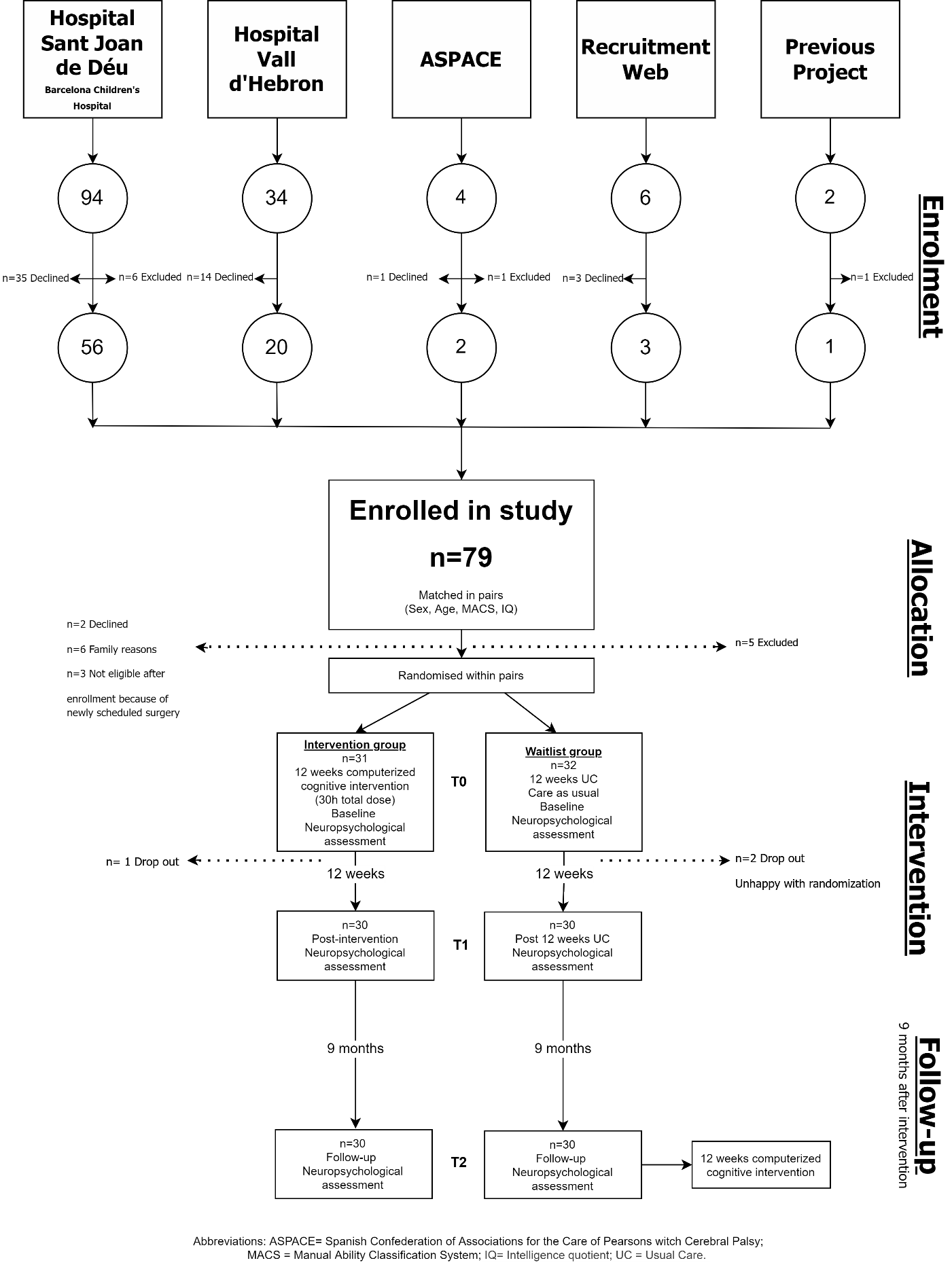


**Figure S1.** Sample flowchart

Abbreviations: IQ = Intelligence Quotient; MACS = Manual Ability Classification System.

**Figure S2**. Graphical representation of differences between intervention and waitlist groups in higher-order graphical representation


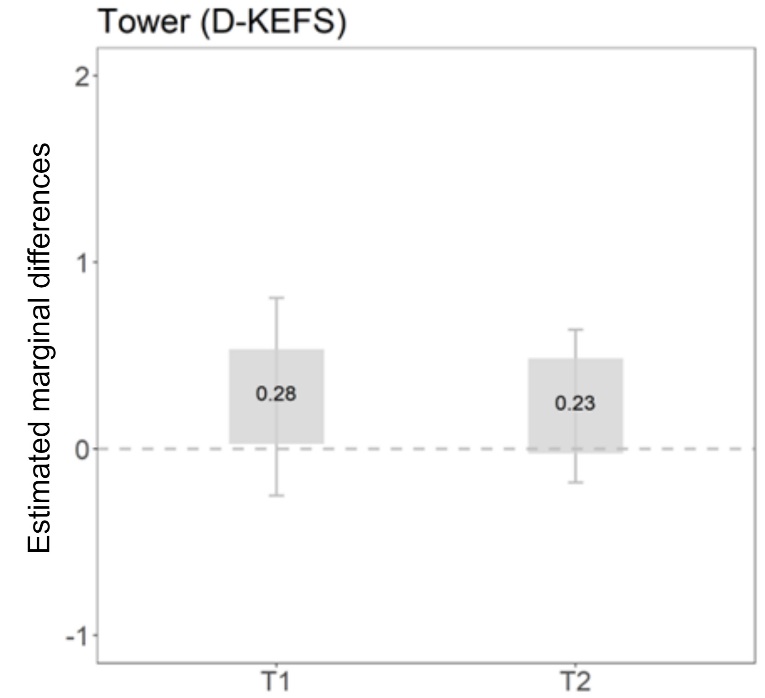


Notes: dark grey box (significant differences between the intervention and waitlist group); light grey (no significant differences). Estimated marginal differences (estimated marginal mean of the intervention group – estimated marginal mean of the waitlist control group) above zero indicate that the intervention group has better performance than the waitlist group. Whiskers correspond to the 95% CIs for the marginal differences.

Abbreviations: T1 = postintervention; T2 = 9-month follow-up after the intervention; D-KEFS = Delis-Kaplan Executive Function System.

**Figure S3**. Graphical representation of differences between intervention and waitlist groups in manifestations of EF in daily life graphical representation


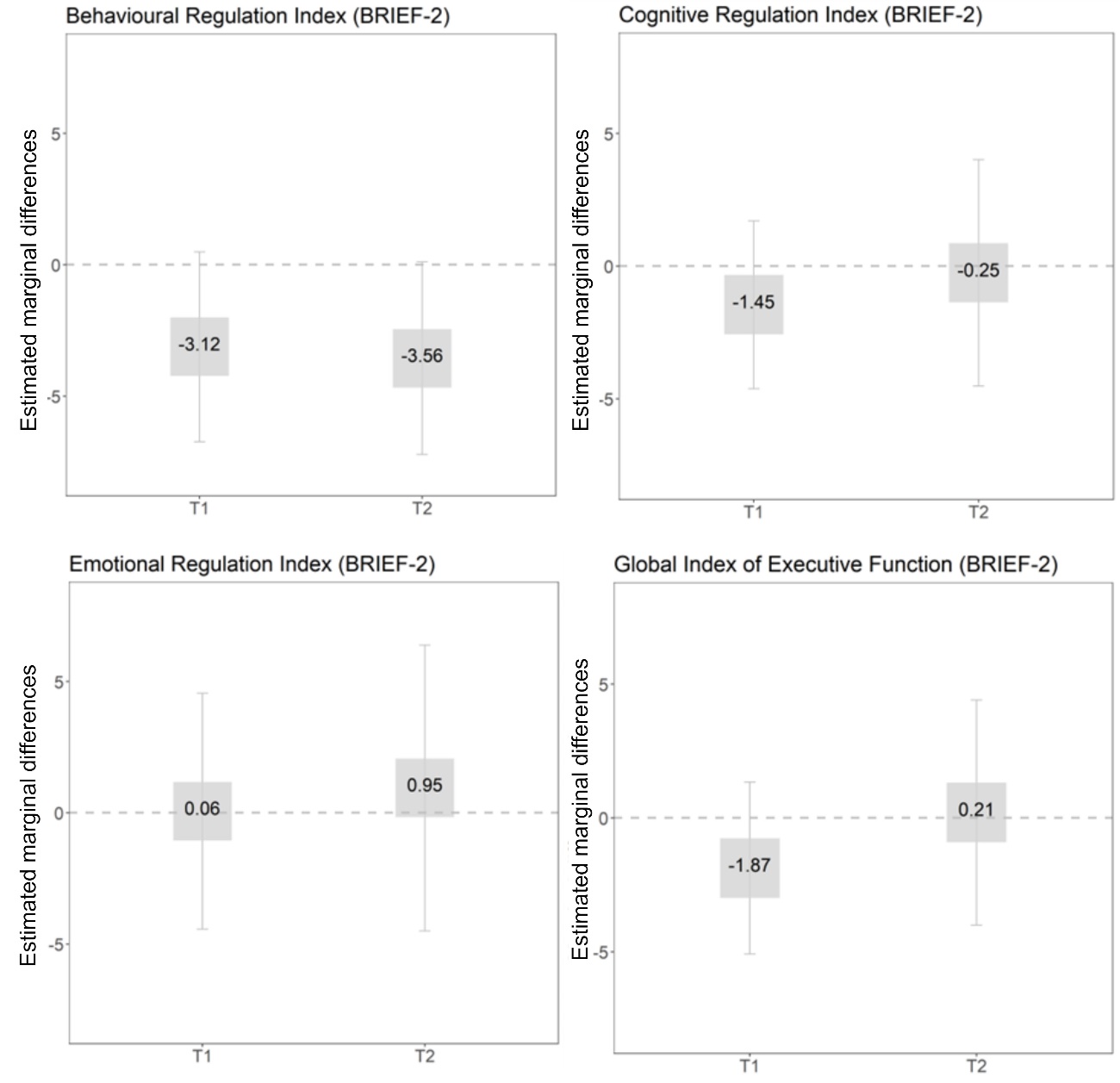


Notes: dark grey box (significant differences between the intervention and waitlist group); light grey (no significant differences). Estimated marginal differences (estimated marginal mean of the intervention group – estimated marginal mean of the waitlist control group) under zero indicate that the intervention group has better performance than the waitlist group. Whiskers correspond to the 95% CIs for the marginal differences.

Abbreviations: T1 = postintervention; T2 = 9-month follow-up after the intervention; BRIEF-2 = Behavior Rating Inventory of Executive Function-2.
